# Nicotinamide Mononucleotide Ameliorates Silica-Induced Lung Injury through the Nrf2-Regulated Glutathione Metabolism Pathway in Mice

**DOI:** 10.3390/nu15010143

**Published:** 2022-12-28

**Authors:** Liqun Wang, Manyu Zhao, Rui Qian, Mengzhu Wang, Qixue Bao, Xuxi Chen, Wen Du, Ling Zhang, Tinghong Ye, Yongmei Xie, Ben Zhang, Lijun Peng, Yuqin Yao

**Affiliations:** 1 Molecular Toxicology Laboratory of Sichuan Provincial Education Office, Institute of Systems Epidemiology, West China School of Public Health and West China Fourth Hospital, Sichuan University, Chengdu 610041, China; 2 West China Occupational Pneumoconiosis Cohort Study (WCOPCS) Working Group, Research Center for Prevention and Therapy of Occupational Disease, West China-PUMC C.C. Chen Institute of Health, West China School of Public Health and West China Fourth Hospital, Sichuan University, Chengdu 610041, China; 3 State Key Laboratory of Biotherapy and Cancer Center, West China Hospital, Sichuan University and Collaborative Innovation Center of Biotherapy, Chengdu 610041, China; 4 Chengdu Chuanyu Jianwei Biotechnology Co., Ltd., Chengdu 610213, China

**Keywords:** silicosis, nicotinamide mononucleotide, oxidative stress, glutathione metabolism, Nrf2

## Abstract

Nicotinamide mononucleotide (NMN) is a natural antioxidant approved as a nutritional supplement and food ingredient, but its protective role in silicosis characterized by oxidative damage remains unknown. In this study, we generated a silicosis model by intratracheal instillation of silica, and then performed histopathological, biochemical, and transcriptomic analysis to evaluate the role of NMN in silicosis. We found that NMN mitigated lung damage at 7 and 28 days, manifested as a decreasing coefficient of lung weight and histological changes, and alleviated oxidative damage by reducing levels of reactive oxygen species and increasing glutathione. Meanwhile, NMN treatment also reduced the recruitment of inflammatory cells and inflammatory infiltration in lung tissue. Transcriptomic analysis showed that NMN treatment mainly regulated immune response and glutathione metabolism pathways. Additionally, NMN upregulated the expression of antioxidant genes *Gstm1*, *Gstm2*, and *Mgst1* by promoting the expression and nuclear translocation of nuclear factor-erythroid 2 related factor 2 (Nrf2). Gene interaction analysis showed that Nrf2 interacted with *Gstm1* and *Mgst1* through *Gtsm2*. Promisingly, oxidative damage mediated by these genes occurred mainly in fibroblasts. In summary, NMN alleviates silica-induced oxidative stress and lung injury by regulating the endogenous glutathione metabolism pathways. This study reveals that NMN supplementation might be a promising strategy for mitigating oxidative stress and inflammation in silicosis.

## 1. Introduction

Silicosis is one of the fastest-growing and most pathogenic major types of pneumoconiosis, accounting for about 39% of cases [1]. Although in 1995 the International Labour Organization and the World Health Organization launched the Global Plan to Eliminate Silicosis by 2030 [2], millions worldwide are still exposed to dangerous levels of silica [3]. Even when working in an environment that meets the exposure limit of 0.05 mg/m^3^ defined by the National Institute of Occupational Health and Safety, the risk of silicosis increases significantly [4]. In 2017, more than 20,000 new cases of silicosis were reported [3]. It therefore can be predicted that the number of silicosis patients will continue to increase, while silicosis remains irreversible and causes more than 10,000 deaths each year [5]. There is an urgent need to find new treatment strategies to delay the course of the disease and improve the quality of life for people suffering from silicosis.

Silica dust deposited in bronchioles and alveolar spaces after inhalation is the predominant risk factor for silicosis. Due to the physical properties of its fine particles, including the surface effect, electrification, and sedimentation [6,7], silica dust is easily adsorbed on the cell surface and damages cells—for example, by inducing reactive oxygen species causing cell stress [8,9]. Under normal physiological conditions, stressed cells secrete signaling molecules, recruit inflammatory cells to activate immune responses, and mobilize macrophages and other phagocytes to remove silica [10]. Subsequently, epithelial cells and fibroblasts are transformed and differentiated in response to cytokines released by activated immune cells, then migrate to injured sites for tissue repair [11]. However, inhalable silica behaves with the physical properties of fine particles that can interact with cells through adhesion and entrapment, and even enter cells [12]. It is difficult for cells to discharge or remove these particles through their own protective mechanisms. Additionally, because the body cannot degrade silica, the phagocytized silica particles can cause cell death, rupture, and dissolution, and are re-released into the tissue microenvironment. This occurs repeatedly through a process of phagocytosis and release, thus constantly causing damage to cells and tissue [13]. In such a case, the injured site usually forms scar tissue due to excessive repair, characterized by inflammatory infiltration and nodules in the early stage of pathology, then further developing into progressive massive fibrosis and continuing to damage lung function. Finally, the patient dies of respiratory failure [11,14]. Based on the pathophysiological process of silicosis, improving stress responses in cells, and alleviating the injury induced by silica are the key measures to slow down the process of silicosis fibrosis.

Nicotinamide mononucleotide (NMN) is a bioactive derivative of vitamin B. As a precursor of nicotinamide adenine dinucleotide (NAD) synthesis, NMN exists in the nucleus, cytoplasm, and mitochondria. Fresh fruits, vegetables, and meat contain NMN, but at relatively low content levels [15]. Clinical studies have found that NMN supplementation could alleviate fatigue and reduce drowsiness in elderly people and enhance insulin sensitivity in pre-diabetic women [16,17]. Studies have shown that in doxorubicin-induced cardiotoxicity, NMN reduced the production of malondialdehyde and reactive oxygen species (ROS), increased levels of glutathione (GSH) and superoxide dismutase activity, and alleviated inflammatory and oxidative damage to the heart [18]. NMN promoted GSH production in a glutathione peroxidase 4-dependent manner and ameliorated oxidative skin damage and lipid peroxidation induced by ultraviolet radiation [19]. Supplementation of NMN attenuated SARS-CoV-2-induced pulmonary inflammation and metabolic disorders [20]. Although many studies have shown that NMN can alleviate inflammation and oxidative damage in pathological conditions, its protective effect on silicosis remains unclear.

In this study, we established a silicosis model and supplemented mice with NMN for 7 d or 28 d, respectively. Histological staining was applied to evaluate the changes in lung tissue structure. Biochemical analysis was carried out to detect the expression of ROS and GSH. Flow cytometry was employed to detect the expression of inflammatory cells. Transcriptome sequencing and single-cell RNA sequencing were conducted to analyze the mechanism of the protective effect afforded by NMN, and molecular biological verification was performed to evaluate the role of NMN in pneumoconiosis injury and oxidative damage.

## 2. Materials and Methods

### 2.1. Reagents and Materials

NMN was manufactured by Chengdu Chuanyu Jianwei Biotechnology Co., Ltd., Chengdu, China (CYJW-003), and the purity of NMN according to assessment by analytical HPLC was higher than 99% (Supplementary Materials File S1). Collagenase I (A004194) and collagenase II (A004174) were purchased from Sangon Biotech, Shanghai, China. Collagenase IV was purchased from BioFroxx, Pfungstadt, Germany (2091). Silica was purchased from Sigma Aldrich, St. Louis, MO, USA (BCBP9096V). APC anti-mouse CD69 antibody (104514), PE anti-mouse CD4 antibody (100408), FITC anti-mouse CD8a antibody (100706), and FITC anti-mouse/human CD11b antibody (101206) were purchased from BioLegend, San Diego, CA, USA, and PE anti-mouse F4/80 antibody was purchased from Invitrogen, Waltham, MA, USA (12-4801-82). The reactive oxygen species assay kit was purchased from KeyGEN BioTECH, Nanjing, China (KGT010-1). Trizol reagent was purchased from Thermo Fisher, Waltham, MA, USA (15596018). Paraformaldehyde (4%) was purchased from Biosharp, Hefei, China (BL539A). Bioanalyzer 2100 and RNA 6000 Nano LabChip kits were purchased from Agilent, Santa Clara, CA, USA (5067-1511). The animal total RNA isolation kit was purchased from FOREGENE, Chengdu, China (RE-03011). Taq Pro Universal SYBR qPCR Master Mix was purchased from Vazyme, Nanjing, China (Q712-02). ABScript III RT Master Mix for qPCR with gDNA remover was purchased from ABclonal, Woburn, MA, USA (RK20429). The reduced glutathione assay kit was purchased from Nanjing Jiancheng Bioengineering Institute, Nanjing, China (A006-2-1). RIPA lysis buffer (P0013B) and phenylmethanesulfonyl fluoride (ST506) were purchased from Beyotime, Shanghai, China; the BCA protein assay kit was purchased from EpiZyme, Cambridge, MA, USA (ZJ101). Anti-Nrf2 rabbit polyclonal antibody (ER1706-41) and HRP conjugated goat anti-mouse IgG goat polyclonal antibody (HA1006) were purchased from HUABIO, Woburn, MA, USA. Anti-β actin mouse antibody (TA-09) and HRP-labeled goat anti-rabbit IgG (H + L) antibody (ZB-2306) were purchased from ZSGB-BIO, Beijing, China.

### 2.2. Animal and Experimental Design

C57BL/6J Nifdc male mice were purchased from Charles River Laboratories (Beijing, China), aged 6~8 weeks, weighing 20 ± 2 g. They were raised in a clean SPF barrier environment, 12–12-h light cycle, room temperature 25 ± 1 °C, humidity 70%, and were free to eat and to drink water, with quarantined adaptive feeding for 2 weeks.

The design scheme of the animal experiments is shown in Table 1. The animals were divided into five groups: the control group (Sham), in which the mice received intratracheal instillation with saline and then daily normal saline via gavage; the model group (Vehicle), where the mice received intratracheal instillation with silica (50 mg/mL, 80 μL) and then daily normal saline by gavage; the high- and low-dose NMN groups (NMN-H and NMN-L, respectively), in which the mice received intratracheal instillation with silica (50 mg/mL, 80 μL), and those in the NMN high-dose group were given 1000 mg/kg NMN daily, and the NMN low-dose group 500 mg/kg NMN daily; and the NMN control group (NMN-Con), in which the mice received intratracheal instillation with silica (50 mg/mL, 80 μL), and then 1000 mg/kg NMN daily. The intratracheal instillation procedures were identical to our previous study [21]. The mice were sacrificed at 7 d or 28 d; the lung tissue was dissected, and the lung weight coefficient was calculated (lung weight coefficient = lung wet weight/body weight × 100). Then, each lobe of the lung was divided into three parts. One part was fixed in 4% paraformaldehyde for histological observation, and the other two were rapidly placed in liquid nitrogen for transcriptome sequencing, biochemical analysis, and molecular biological analysis.

### 2.3. Flow Cytometry

Preparation of single-cell suspension: a twenty milligram sample lung tissue was cut and digested using collagenase (1 mg/mL collagenase IV, dissolved with serum-free medium) for 1.5 h (37 °C). After centrifugation, the precipitate was washed three times with PBS and then resuspended with 1 mL of PBS. After standing for 1 min, 300 μL of cell suspension was used for subsequent experiments.

ROS detection: a DCFH-DA probe solution was employed to detect the expression of ROS. DCFH-DA was diluted with a serum-free medium at the ratio of 1:700, then 700 μL of diluted DCFH-DA probe was added to the single-cell lung tissue suspension, and cells were stained with 10 μmol/L DCFH-DA probe for 20 min at 37 °C in the dark. Next, cells loaded with probe solution were washed three times with serum-free medium and resuspended in 0.5 mL of serum-free medium, and ROS levels were determined within 1 h using flow cytometry (Novocyte, San Diego, CA, USA). The mean fluorescence intensity was used for statistical analysis.

Detection of macrophages, CD4 + CD69 + T cells, and CD8 + CD69 + T cells: one microliter of 0.2 mg/mL antibody was added to the single-cell suspension to label macrophages, CD4 + CD69 + T cells, and CD8 + CD69 + T cells, respectively. The cells were dyed in the dark at room temperature for 30 min, washed twice with PBS, and resuspended with 0.5 mL PBS. Macrophages were labeled with PE anti-mouse F4/80 and FITC anti-mouse/human CD11b antibodies; CD4 + CD69 + T cells were labeled with APC anti-mouse CD69 and PE anti-mouse CD4 antibodies; and CD8 + CD69 + T cells were labeled with APC anti-mouse CD69 and FITC anti-mouse CD8a antibodies.

### 2.4. Histopathological Staining

After 72 h of fixation in 4% paraformaldehyde, the tissues were washed overnight in running water, dehydrated, embedded in paraffin, and sliced into 3–5 μm sections. After dewaxing and hydration, hematoxylin and eosin (H&E), Masson staining, and immunohistochemical staining were performed, respectively, with steps the same as in our previous study [22]. The dilution ratio of Nrf2 antibody used for the immunohistochemical staining was 1:500. Pathological scores were performed according to Ashcroft’s method [23]. In brief, we created pathology scores using modified Ashcroft’s criteria (grades 0–8) for 9 different fields (magnification: 10×) from at least 3 animals. For Masson staining analysis, the collagen fibers’ staining area and the total area of each field (magnification: 10×) were analyzed using Image J software (National Institutes of Health, Bethesda, MD, USA), then we calculated the collagen volume fraction (collagen volume fraction = area of collagen fibers’ staining/total area of the field × 100%).

### 2.5. RNA Sequencing

About 30 mg lung tissue was used for RNA extraction. Total RNA was extracted using Trizol reagent following the manufacturer’s procedure. The total RNA quantity and purity were analyzed using a Bioanalyzer 2100 and RNA 6000 Nano LabChip kit, and high-quality RNA samples with RIN number >7.0 were employed to construct the sequencing library. After quality inspection, RNA was reverse transcribed to establish the cDNA library. Then, the 2 × 150 bp paired-ended sequencing (PE150) was performed on an Illumina Novaseq™ 6000 (LC-Bio Technology CO., Ltd., Hangzhou, China) following the vendor’s recommended protocol. Differential gene expression analysis, gene ontology (GO) analysis, and Kyoto Encyclopedia of Genes and Genomes (KEGG) analysis were performed. Sequencing and bioinformatics analysis were completed by LC-Bio Technology Co., Ltd. (Hangzhou, China).

### 2.6. Single-Cell RNA Sequencing

Lung tissue was separated into single cells using collagenase (0.5 mg/mL collagenase I and 0.5 mg/mL collagenase II, 20~30 min, 37 °C), labeled with a specific barcode and unique molecular identifier, and single cells were sequenced using the 10× genomics platform based on the micro droplet method. Cell Ranger 6.1.2 was employed to filter data and analyze gene expression, and Seurat 4.1.0 to filter cells and analyze cell subtypes. Visual analysis was performed using OmicStudio tools (https://www.omicstudio.cn, accessed on 3 July 2021, LC-Bio Technology Co., Ltd.).

### 2.7. Quantitative Real-Time Polymerase Chain Reaction (qRT-PCR)

About 10 mg lung tissue was weighed and total RNA was extracted using the animal total RNA isolation kit. The operation steps were carried out according to the kit instructions. After quantification, RNA was reverse-transcribed with a reverse-transcription kit, and qRT-PCR was performed using Taq Pro Universal SYBR qPCR Master Mix. The primers used in qRT-PCR are listed in Table 2. β-actin was used as an internal control. The relative mRNA expression level was calculated using the 2^−ΔΔCT^ method.

### 2.8. GSH Detection

The content of GSH in lung tissue was measured using a reduced glutathione assay kit. In brief, approximately 20 mg lung tissue was weighed, added to normal saline (μL) at a ratio of 1:9 and homogenized for 30 s. After centrifugation (2500 r/min, 10 min), the supernatant was collected for detection according to the instructions. The OD value at 405 nm was measured using a microplate reader (Multiskan Go, Thermo Scientific, Waltham, MA, USA), and the level of GSH in the lung tissue was calculated.

### 2.9. Western Blot

A total of 15 mg lung tissue was homogenized by RIPA lysis for 30 min at 4 °C. Total protein was quantified using a BCA kit, and 30–50 μg protein was separated using 10% SDS-PAGE gel and transferred to the polyvinylidene fluoride membrane. Then, the membranes were blocked with 5% skim milk (dissolved in TBST buffer solution) at room temperature for 1 h and incubated with primary antibody overnight at 4 °C (Nrf2 1:500; β-actin 1:1000). After washing with TBST buffer, the membranes were incubated with HRP-conjugated goat anti-mouse IgG goat polyclonal antibody (at a dilution ratio of 1:50,000) and HRP-labeled goat anti-rabbit IgG (H + L) antibody (at a dilution ratio of 1:3000), respectively. Then, the membranes were visualized using Invitrogen iBright imaging systems (Thermo Fisher, Waltham, MA, USA).

### 2.10. Data Statistics and Analysis

The data are presented as the mean ± standard error of mean from at least three biological replicates. Microscopic images of H&E and Masson staining were analyzed using the Ashcroft scores and the collagen volume fraction, respectively. Graph Prism 9.0 (Graphpad, San Diego, CA, USA) and Microsoft Excel 2019 (Microsoft, Redmond, WA, USA) were used for statistical analysis. One-way analysis of variance was carried out to compare the statistical differences between multiple groups and Student *t* testing to compare the statistical differences between two groups, where ^#^ or * indicates *p* < 0.05, ^##^ or ** *p* < 0.01, ^###^ or *** *p* < 0.001, and ^####^ or **** *p* < 0.0001. Finally, Photoshop 2021 software (Adobe Systems, San Jose, CA, USA) was utilized to prepare the figures.

## 3. Results

### 3.1. NMN Alleviates Silica-Induced Lung Injury

**Figure 1:**
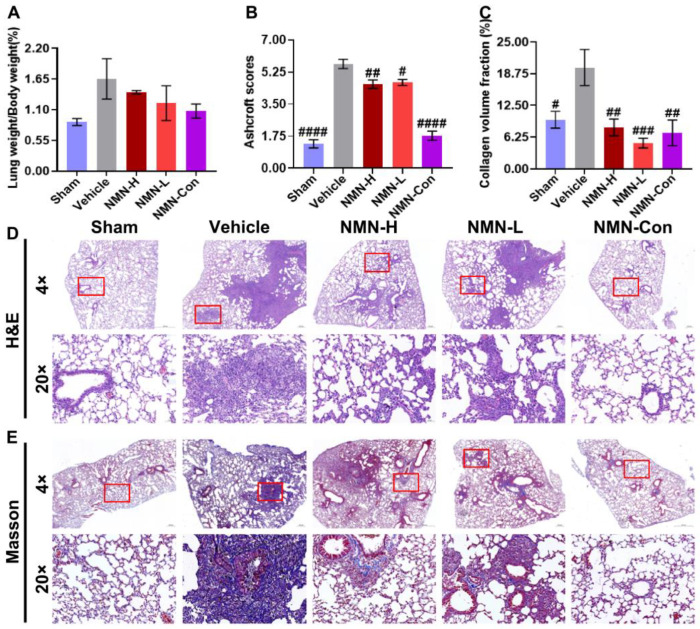
NMN intervention for 7 days ameliorates silica-induced lung injury in mice. (**A**) Lung weight coefficient (the ratio of lung wet weight to body weight) increased during injury induced by silica; (**B**) Ashcroft scores of lung tissue—associated with the severity of lung lesions— increased after silica induction; (**C**) collagen volume fraction of lung tissue—an indicator of collagen fibers’ expression in tissues—where silica induced deposition of collagen fibers in lung tissues; (**D**) H&E staining images of lung tissue; (**E**) Masson staining images of lung tissue. Scale: 4×, 20×, the images for 20× are derived from the red box in 4×. # indicates *p* < 0.05, ## *p* < 0.01, ### *p* < 0.001, #### *p* < 0.0001 vs. Vehicle. Sham: saline + saline; Vehicle: silica + saline; NMN-H: silica + NMN (1000 mg/kg); NMN-L: silica + NMN (500 mg/kg); NMN-Con: saline + NMN (1000 mg/kg).

To investigate the protective effect of NMN against silicosis, we established a mouse silicosis model via intratracheal instillation of a silica suspension. Seven days after exposure to silica, compared with the Sham group, the lung weight coefficient, pathological score, and collagen volume fraction of the Vehicle group had increased (Figure 1A–C), suggesting that lung injury occurred in those mice. H&E staining showed that silica exposure caused inflammatory infiltration and cell proliferation in lung tissue, and the alveolar structure was destroyed, with collagen fibers significantly deposited in the proliferative cell space. In contrast, the Sham group maintained a normal pulmonary architecture with thin-lined alveolar septa (Figure 1D,E). NMN decreased the lung weight coefficient of mice and significantly diminished pathological scores and the fraction of collagen volume. Histologically, NMN treatment reduced inflammatory cell infiltration in lung tissue. Compared with the massive cell proliferation in the Vehicle group, severity levels of lung damage in the NMN-H group and the NMN-L group were low. The NMN-H group did not form obvious large inflammatory cell nodules, with only local cell proliferation around the trachea, and the alveolar structure was relatively complete, indicating that short-term NMN intervention reduced the silica-induced lung injury.

**Figure 2:**
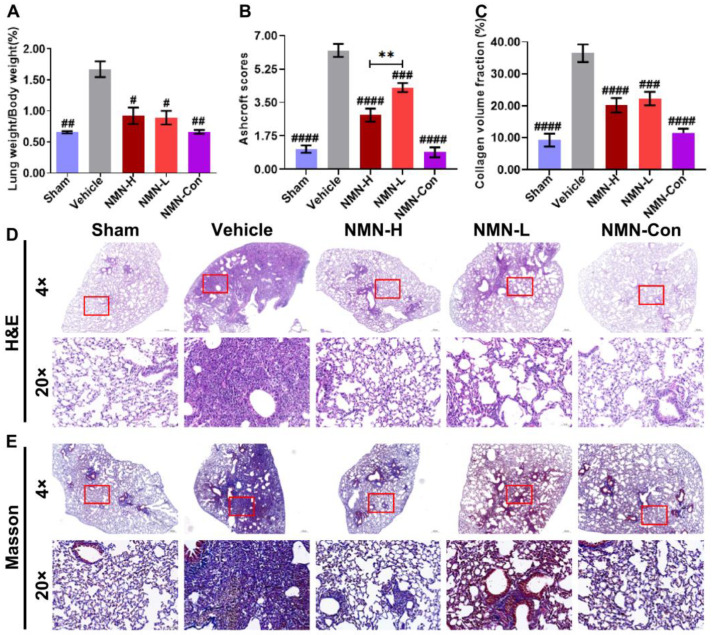
NMN intervention for 28 days ameliorates silica-induced lung injury in mice. (**A**) Lung weight coefficient (the ratio of lung wet weight to body weight) increased during injury induced by silica; (**B**) Ashcroft scores of lung tissue—associated with the severity of lung lesions—increased after silica induction; (**C**) collagen volume fraction of lung tissue—an indicator of collagen fibers expression in tissues—where silica induced deposition of collagen fibers’ in lung tissues; (**D**) H&E staining images of lung tissue; (**E**) Masson staining images of lung tissue. Scale: 4×, 20×, the images for 20× are derived from the red box in 4×. # indicates *p* < 0.05, ## *p* < 0.01, ### *p* < 0.001, #### *p* < 0.0001 vs. Vehicle; ** *p* < 0.01, NMN-H vs. NMN-L. Sham: saline + saline; Vehicle: silica + saline; NMN-H: silica + NMN (1000 mg/kg); NMN-L: silica + NMN (500 mg/kg); NMN-Con: saline + NMN (1000 mg/kg).

After 28 d of silica infusion, the lung weight coefficient of mice had significantly increased; the pathological scores and collagen volume fraction were significantly greater; the lung tissue structure was seriously damaged, accompanied by diffuse cell proliferation; and the deposition of collagen fibers in the interstitial tissue had significantly increased (Figure 2A–E). Similar to the results for 7 d, NMN supplementation significantly attenuated lung injury, cell proliferation, and collagen deposition induced by silica. At 28 d, compared with the Vehicle group, the lung tissue structure of the NMN-H group was relatively complete, with only a few small inflammatory cell nodules. The degree of lung injury in the NMN-L group was also lower than that in the Vehicle group, suggesting that NMN continuously alleviates silica-induced lung injury.

### 3.2. NMN Mitigates Silica-Induced Lung Oxidative Stress and Inflammation

**Figure 3:**
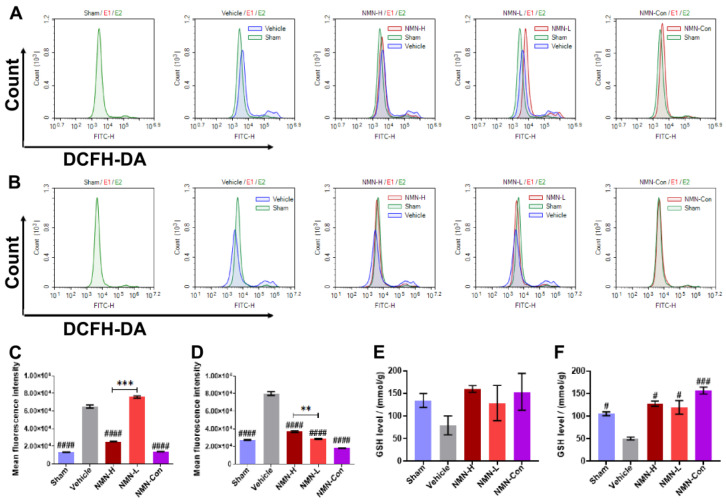
NMN intervention regulates the expression of ROS and GSH in silica-induced lung injury. (**A**) Histogram of ROS levels from flow cytometry after exposure to silica for 7 d; (**B**) histogram of ROS levels from flow cytometry after exposure to silica for 28 d; (**C**) expression of ROS in lung tissue at 7 d, analyzed using mean fluorescence intensity from flow cytometry; (**D**) expression of ROS in lung tissue at 28 d, analyzed using mean fluorescence intensity from flow cytometry; (**E**) expression of GSH in lung tissue at 7 d; (**F**) expression of GSH in lung tissue at 28 d. # indicates *p* < 0.05, ### *p* < 0.001, #### *p* < 0.0001 vs. Vehicle; ** *p* < 0.01, *** *p* < 0.001, NMN-H vs NMN-L. ROS: reactive oxygen species, GSH: glutathione. Sham: saline + saline; Vehicle: silica + saline; NMN-H: silica + NMN (1000 mg/kg); NMN-L: silica + NMN (500 mg/kg); NMN-Con: saline + NMN (1000 mg/kg).

Oxidative stress is a proven mechanism for lung tissue damage after silica exposure. The small exogenous particles induce excessive ROS release in the tissue and destroy the redox balance, leading to oxidative damage in cells in the tissue. Therefore, we applied flow cytometry to detect the level of ROS in lung tissue. Compared with the Sham group, levels of ROS in lung tissue increased obviously after exposure to silica for 7 d and 28 d (Figure 3A–D), suggesting that silica continued to induce severe oxidative stress in lung tissue. At 7 d, 1000 mg/kg NMN supplementation significantly reduced the level of ROS, while 500 mg/kg NMN did not seem to have a protective effect. However, levels of ROS in the lung tissue of mice in the NMN-H and NMN-L groups were effectively decreased at 28 d, and 500 mg/kg NMN reduced ROS to a lower level than in the NMN-H group. GSH is one of the main substrates for ROS scavenging, and we therefore also measured GSH levels in the lung tissue. Compared with the Sham group, the expression of GSH in lung tissue decreased after silica infusion, while the expression of GSH in lung tissue increased in a dose-dependent manner with NMN (Figure 3E,F), demonstrating that NMN alleviates silica-induced lung oxidative damage.

**Figure 4:**
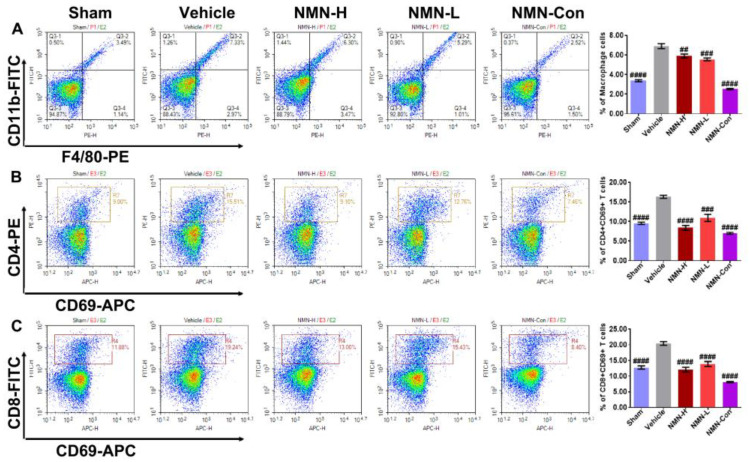
NMN intervention for 7 days decreases the proportion of macrophages, CD4 + CD69 + T cells, and CD8 + CD69 + T cells in silica-induced lung injury. (**A**) Proportions of macrophages in lung tissue; (**B**) proportions of CD4 + CD69 + T cells in lung tissue; (**C**) proportions of CD8 + CD69 + T cells in lung tissue. ## indicates *p* < 0.01, ### *p* < 0.001, #### *p* < 0.0001 vs. Vehicle). Sham: saline + saline; Vehicle: silica + saline; NMN-H: silica + NMN (1000 mg/kg); NMN-L: silica + NMN (500 mg/kg); NMN-Con: saline + NMN (1000 mg/kg).

**Figure 5:**
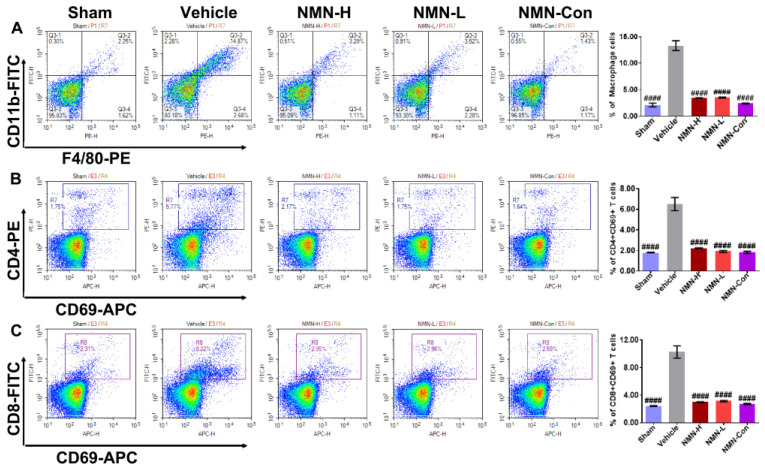
NMN intervention for 28 days decreases the proportion of macrophages, CD4 + CD69 + T cells, and CD8 + CD69 + T cells in silica-induced lung injury. (**A**) Proportions of macrophages in lung tissue; (**B**) proportions of CD4 + CD69 + T cells in lung tissue; (**C**) proportions of CD8 + CD69 + T cells in lung tissue. #### indicates *p* < 0.0001 vs. Vehicle. Sham: saline + saline; Vehicle: silica + saline; NMN-H: silica + NMN (1000 mg/kg); NMN-L: silica + NMN (500 mg/kg); NMN-Con: saline + NMN (1000 mg/kg).

The body has a self-repair mechanism. During oxidative damage, the body can release damage signals to induce an immune response to correct the damage and the released damage signals can recruit inflammatory cells to the damage site. During the progression of silicosis, failure to eliminate silica particles can cause continuous damage to lung tissue, leading to excessive recruitment of immune cells and aggravating the inflammatory injury. Therefore, using flow cytometry we examined the proportion of inflammatory cells including macrophages, CD4 + CD69 + T cells, and CD8 + CD69 + T cells in lung tissue. Silica rapidly induced abnormal increases in proportions of macrophages, CD4 + CD69 + T cells, and CD8 + CD69 + T cells at 7 d (Figure 4A–C). At 28 d, these anomalies persisted (Figure 5A–C). These results suggest that silica can disrupt the immune balance of lung tissue, resulting in inflammatory injury. Doses of both 500 mg/kg and 1000 mg/kg NMN significantly reduced the proportions of macrophages, CD4 + CD69 + T cells, and CD8 + CD69 + T cells in lung tissue after silica infusion. On the 28th day, the proportions of these immune cells in the NMN-H and NMN-L groups were almost restored to the same level as in the Sham group, suggesting that NMN alleviates silica-induced inflammatory injury and corrects immune disorder in lung tissue.

### 3.3. NMN Suppresses Silica-Induced Lung Injury through the Glutathione Metabolism Pathway

**Figure 6:**
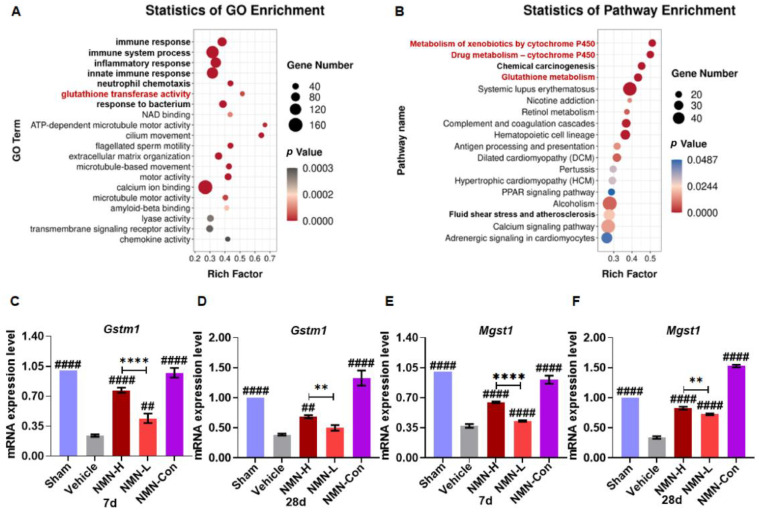
NMN protects against silica-induced lung injury through immune response and GSH-related metabolism pathways. (**A**) GO analysis of lung tissue transcriptomic sequencing; (**B**) KEGG analysis of 30 differentially expressed genes from GO analysis; (**C**) relative expression of *Gstm1* mRNA in lung tissue at 7 d; (**D**) relative expression of *Gstm1* mRNA in lung tissue at 28 d; (**E**) relative expression of *Mgst1* mRNA in lung tissue at 7 d; (**F**) relative expression of *Mgst1* mRNA in lung tissue at 28 d. ## indicates *p* < 0.01, #### *p* < 0.0001 vs. Vehicle. ** *p* < 0.01, **** *p* < 0.0001, NMN-H vs NMN-L. Sham: saline + saline; Vehicle: silica + saline; NMN-H: silica + NMN (1000 mg/kg); NMN-L: silica + NMN (500 mg/kg); NMN-Con: saline + NMN (1000 mg/kg).

To further analyze the protective mechanism of NMN against silicosis, we performed transcriptomic sequencing analysis of lung tissues after 28 days of silica exposure. GO analysis showed that the protective effects of NMN against silicosis were mainly related to immune response, ATP metabolism, NAD binding, and glutathione transferase (Figure 6A). The top 10 GO terms of biological process, cellular component, and molecular function were analyzed, and 30 differentially expressed genes were obtained (*p* < 0.05, Supplementary Table S1). KEGG analysis showed that these genes were mainly enriched in 54 KEGG signaling pathways (Supplementary Table S2), of which 18 KEGG signaling pathways showed significant changes (*p* < 0.05) involving cytochrome P450 (CYP450) metabolism, glutathione metabolism, retinol metabolism, the PPAR signaling pathway, and calcium metabolism (Figure 6B). Among them, *Gstm1*, *Gsto1*, *Gsta4*, and *Mgst1* jointly participate in the regulation of multiple signaling pathways with significant effects, including the metabolism of xenobiotics by cytochrome P450 (ko00980, *p* < 0.001), drug metabolism by cytochrome P450 (ko00982, *p* < 0.001), chemical carcinogenesis (ko05204, *p* < 0.001), glutathione metabolism (ko00480, *p* < 0.001), fluid shear stress and atherosclerosis (ko05418, *p* < 0.05). Three genes were related to glutathione metabolism. Furthermore, qRT-PCR detection showed that silica significantly decreased the expression of *Gstm1*, *Mgst1*, and *Gsta4*, although not *Gsto1* (Figure 6C–F and Figure S1). At 7 d and 28 d, 1000 mg/kg NMN significantly restored the expression of *Gstm1* and *Mgst1*.

**Figure 7:**
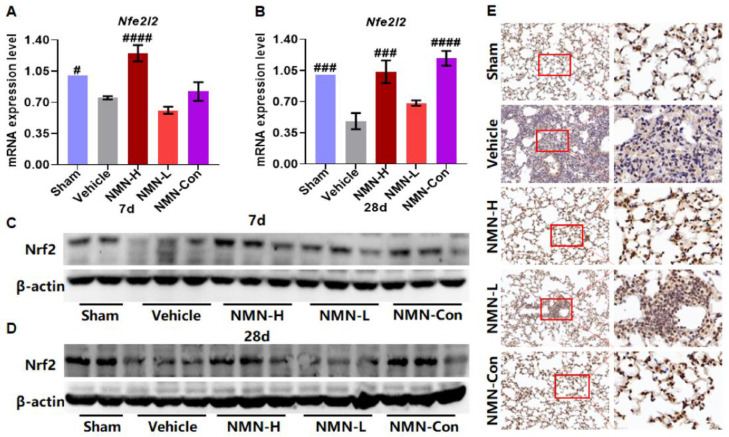
NMN regulates the expression of Nrf2 in silica-induced lung injury. (**A**) The relative expression of *Nfe2l2* mRNA in lung tissue at 7 d; (**B**) relative expression of *Nfe2l2* mRNA in lung tissue at 28 d; (**C**) expression of Nrf2 protein in lung tissue at 7 d; (**D**) expression of Nrf2 protein in lung tissue at 28 d; (**E**) expression and distribution of Nrf2 in lung tissue detected by immunohistochemical staining. Scale: 20×, 63×. # indicates *p* < 0.05, ### *p* < 0.001, #### *p* < 0.0001 vs. Vehicle. Sham: saline + saline; Vehicle: silica + saline; NMN-H: silica + NMN (1000 mg/kg); NMN-L: silica + NMN (500 mg/kg); NMN-Con: saline + NMN (1000 mg/kg).

Nrf2 is the key factor that regulates GSH production and redox balance, and *Gstm1* and *Mgst1* are the target genes of Nrf2. Therefore, we further detected the expression of Nrf2 mRNA (*Nfe2l2*) and Nrf2 protein in lung tissue. Silica reduced the expression of *Nfe2l2* and Nrf2 (Figure 7A–D), while the expression of *Nfe2l2* and Nrf2 was upregulated with NMN treatment. As shown in Figure 7E, we observed Nrf2 present in the cytoplasm in the Vehicle group, and translocated into the nucleus after NMN supplementation, suggesting that NMN may regulate the expression of antioxidant-related genes by promoting Nrf2 expression and nuclear translocation.

**Figure 8:**
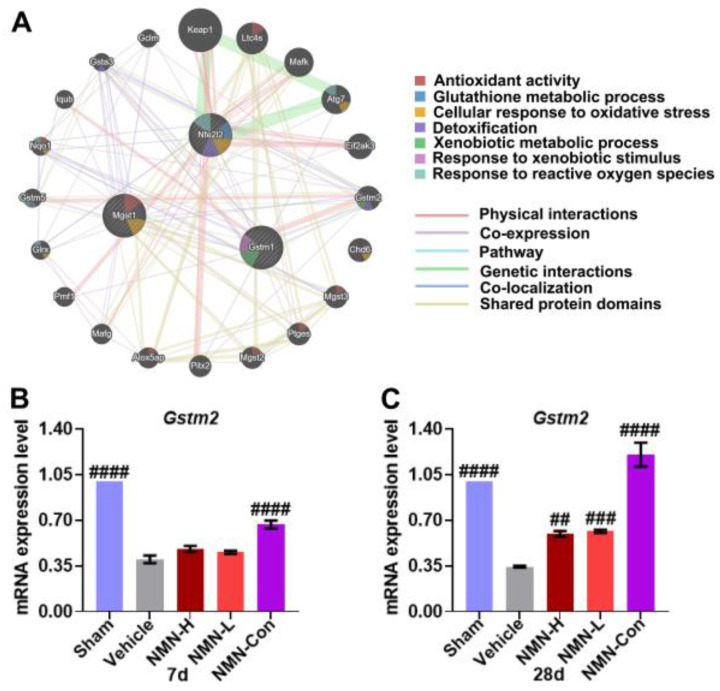
*Gstm1*, *Mgst1*, and *Nfe2l2* gene interaction analysis and the expression of Gstm2 in silica-induced lung injury. (**A**) *Gstm1*, *Mgst1*, and *Nfe2l2* gene–gene interaction network analysis from GeneMANIA database, *Nfe2l2* interacts with *Gstm1* and *Mgst1* genes via *Gstm2*; (**B**) relative expression of *Gstm2* mRNA in lung tissue at 7 d; (**C**) relative expression of *Gstm2* mRNA in lung tissue at 28 d. ## indicates *p* < 0.01, ### *p* < 0.001, #### *p* < 0.0001 vs. Vehicle. Sham: saline + saline; Vehicle: silica + saline; NMN-H: silica + NMN (1000 mg/kg); NMN-L: silica + NMN (500 mg/kg); NMN-Con: saline + NMN (1000 mg/kg).

To further investigate the relationship between Nrf2 and GSH metabolism in silicosis, we used the GeneMANIA database (http://genemania.org/, accessed on 3 July 2021) to analyze the gene interaction network of *Gstm1*, *Mgst1*, and *Nfe2l2* and found that these three genes form a gene–gene interaction network through *Gstm2* (Figure 8A). The interaction between *Gstm1* and *Mgst1* was co-localization. *Gstm1* interacts with *Gstm2* through physical interactions, co-expression, and co-localization, and they share protein domains. *Mgst1* was co-localized with *Gstm2*. *Nfe2l2* was co-expressed with the *Gstm2* gene. *Nfe2l2*, *Gstm1*, and *Mgst1* had no direct interaction. Functional analysis showed that *Gstm1* and *Gstm2* were mainly related to the response to xenobiotic stimulus, the xenobiotic metabolic process, and the detoxification process, and the main function was to react and metabolize exogenous substances. *Nfe2l2* was related to the response to reactive oxygen species, cellular response to oxidative stress, the glutathione metabolic process, and detoxification, which may respond to ROS and oxidative stress, regulate GSH metabolism, and promote detoxification, respectively; *Mgst1* was related to cellular response to oxidative stress and antioxidant activity, and its main function is to respond to oxidative stress and exert antioxidant activity. Given the role of *Gstm2* among *Gstm1*, *Mgst1*, and *Nfe2l2* genes, we further examined the expression of *Gstm2* in lung tissue. The results showed that expression of *Gstm2* was also downregulated after silica exposure, and NMN supplementation increased the expression of *Gstm2* at 28 d (Figure 8B,C). These results suggest that NMN promotes GSH synthesis by upregulating the expression of antioxidant genes to scavenge ROS and alleviates lung oxidative damage.

### 3.4. Silica Induces Glutathione Metabolism Disorder in Fibroblasts

**Figure 9:**
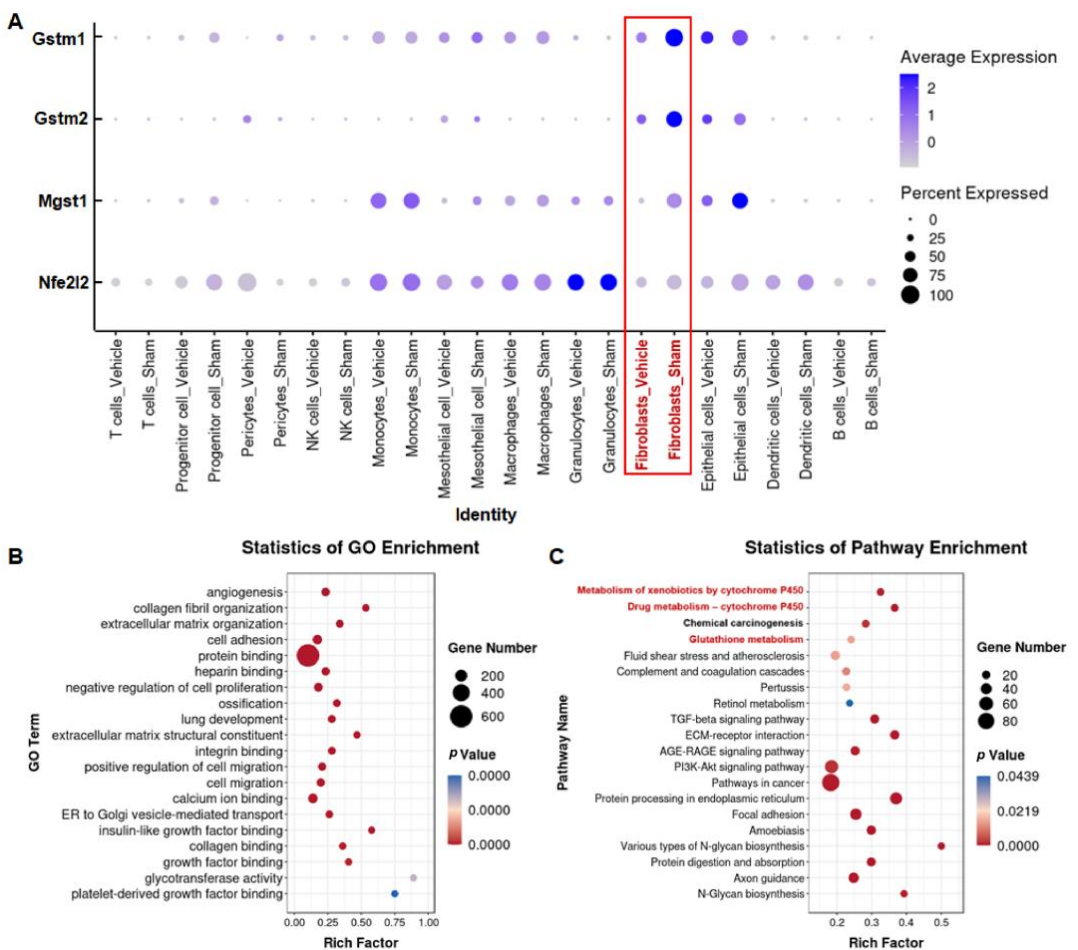
Silica induces oxidative imbalance in fibroblasts. (**A**) The relative expression levels of *Gstm1*, *Gstm2*, *Mgst1*, and *Nfe2l2* in different types of lung cells after silica exposure, detected by single-cell RNA sequencing. (**B**) GO analysis of fibroblasts performed using single-cell sequencing data; (**C**) KEGG analysis of fibroblasts performed using single-cell sequencing data.

To further explore the potential mechanism of NMN protection against silica-induced lung injury, we used single-cell RNA sequencing data to analyze the expression of *Gstm1*, *Gstm2*, *Mgst1*, and *Nfe2l2* in different types of lung cells. Compared with the Sham group, the expression of antioxidant-related genes in lung tissue was significantly decreased after silica induction (Supplementary Figure S2). *Gstm1*, *Gstm2*, *Mgst1*, and *Nfe2l2* were co-expressed mainly in fibroblasts, and the expression level decreased significantly after silica exposure (Figure 9A and Figure S3). GO analysis showed that the main biological processes and molecular functions were collagen fibril organization (GO:0030199, *p* < 0.001), extracellular matrix organization (GO:0030198, *p* < 0.001), and extracellular matrix structural constituents (GO:0005201, *p* < 0.001) (Figure 9B), reflecting the typical functional characteristics of fibroblasts. KEGG enrichment analysis showed that signaling pathways related to oxidative stress, such as metabolism of xenobiotics by cytochrome P450 (ko00980, *p* < 0.001), drug metabolism by cytochrome P450 (ko00982, *p* < 0.001), and glutathione metabolism (ko00480, *p* < 0.05) changed significantly in fibroblasts, similar to results observed in our transcriptomic studies (Figure 6B and Figure 9C). Concomitantly, the classical signaling mechanisms related to fibrosis also changed significantly in fibroblasts, including the TGF-beta signaling pathway (ko04350, *p* < 0.001), PI3K-Akt signaling pathway (ko04151, *p* < 0.001), and extracellular matrix (ECM)–receptor interaction (ko04512, *p* < 0.001), suggesting that endogenous oxidative imbalance mediated by *Gstm1*, *Gstm2*, *Mgst1*, and *Nfe2l2* may occur mainly in fibroblasts and is accompanied by pro-fibrotic changes.

## 4. Discussion

Silicosis is a rapidly progressing and incurable disease, and oxidative damage is an important component of silicosis, characterized by elevated levels of ROS and depleted GSH [24,25]. Therefore, alleviating oxidative damage is an effective measure to delay the process of silicosis. In this study, we found that NMN ameliorated silica-induced oxidative damage in silicosis by reducing inflammatory cell infiltration, decreasing ROS production, and promoting GSH synthesis. These effects were achieved mainly by regulating the metabolism of xenobiotics by the cytochrome P450 and glutathione metabolic pathways, which are jointly regulated by antioxidant genes such as *Nfe2l2*, *Gstm1*, *Gstm2*, and *Mgst1*. These results indicate that NMN might be an effective antioxidant to alleviate silicosis and other lung disease induced by silica.

Oxidative damage and inflammatory injury are early events in many fibrotic diseases and persist throughout the course of the disease [24,26]. We found that NMN continuously reduced ROS levels in lung tissue, and alleviated oxidative damage induced by silica exposure. Previous studies have indicated that NMN is associated with similar responses in UVB-induced skin damage [19]. During injury, due to its self-regulation ability the body induces immune responses to enable repair. However, silica is a special xenobiotic that cannot be completely removed when it enters the alveoli, and it continues to experience phagocytosis and release, causing repeated harm to lung tissue [13]. This immune response persists and prompts the recruitment and infiltration of inflammatory cells in the lung tissue, in turn causing inflammatory damage. Macrophages are the main phagocytes in lung tissue and are an important cell types in inflammatory injury in lung tissue. Studies have shown that NMN significantly inhibited the activation of macrophages [27], and we found that NMN intervention significantly reduced macrophage recruitment, suggesting that NMN could alleviate inflammatory damage by regulating the homeostasis of macrophages. In previous studies, infiltration by CD4 + T cells and CD8 + T cells have been observed in silicosis [28]. We found that the numbers of CD4 + CD69 + T cells and CD8 + CD69 + T cells increased significantly after silica exposure. CD69 is a classic marker of T-cell activation [29], and T cells with high CD69 expression promote the progression of inflammatory diseases and fibrosis [30,31]. Previous studies have also reported increases in the expression of CD69 in lymphocytes after silica exposure [32], indicating that T lymphocytes are in an active immune-excited state, and this may aggravate inflammatory infiltration in lung tissue. NMN supplementation reduced the proportion of activated CD4+ and CD8+ T cells, suggesting that NMN has therapeutic potential for treatment of silica-induced lung inflammation. Our transcriptomic results support this view, with GO analysis showing that NMN intervention resulted primarily in significant changes in immune response, inflammatory response, innate immune response, and other immune-related biological processes. Additionally, a recent study on SARS-CoV-2 pneumonia showed that NMN supplementation reduced the infiltration of inflammatory cells in lung tissue and the expression of inflammatory-related genes [20], further supporting our conclusion that NMN plays a protective role in silica-induced oxidative and inflammatory damage.

Similar to immune regulation, the body also has an endogenous antioxidant defense system, and glutathione is an important protective mediator within this defense system [33]. Based on transcriptomics analysis, we found that the signaling mechanisms were related to endogenous antioxidant systems, such as the P450 metabolic pathway and glutathione metabolic pathway, which changed significantly after silica exposure, further indicating that silica caused oxidative damage in lung tissue and triggered an imbalance in the endogenous redox metabolic mechanism. Previous metabolomic studies have found that glutathione metabolism was significantly disturbed by exposure even to low doses of silica [34]. Our study showed that the co-expressed genes *Gstm1*, *Gstm2*, and *Mgst1* in the P450 metabolic pathway and glutathione metabolic pathway were significantly downregulated after silica induction, and studies indicated that a lack of *Gstm1* and *Mgst1* genes led to ROS production and aggravated inflammatory response [35,36]. NMN upregulated the expression of *Gstm1* and *Mgst1*, demonstrating its ability to reduce ROS production and alleviate inflammation. Nrf2 is the main regulator of redox homeostasis and is also a co-regulator of *Gstm1*, *Gstm2*, and *Mgst1* [37,38]. In this study, we observed that gene and protein expression of Nrf2 decreased in lung tissue after silica exposure, which may account for ROS accumulation, downregulation of antioxidant genes, and associated oxidative damage. We found that NMN supplementation restored the nuclear transfer of Nrf2 and effectively upregulated the expression of these antioxidant genes. Recent studies have also shown that NMN can upregulate the expression of Nrf2 and enhance the antioxidant ability of intestinal cells [39]. These results suggest that the protective effect of NMN against oxidative stress is regulated by Nrf2.

Glutathione S-transferase polymorphisms are associated with susceptibility to many lung diseases [40]. Asbestosis patients with *Gstm1* deletion have a greater pleural scarring and higher risk of pulmonary parenchymal disease due to a gene-deletion-induced decrease in detoxification ability [41]. Transfecting extracellular vesicles with high *Gstm2* expression in fibroblasts enhanced antioxidant capacity, inhibited lipid peroxidation, and maintained their normal function [42], while Mgst1 overexpression protected cells from oxidative damage by regulating calcium loading capacity [43]. In the current study, we found that NMN attenuated silica-induced lung injury by upregulating the expression of *Nfe2l2*, *Gstm1*, *Gstm2*, and *Mgst1*. We used single-cell sequencing data to analyze the expression of these genes in different cell types, to explore which cell types are most likely to benefit from this protective effect. We found that mainly *Nfe2l2*, *Gstm1*, *Gstm2*, and *Mgst1* genes were co-expressed in fibroblasts, mesothelial cells, and epithelial cells. In fibroblasts, the expression of these genes was significantly downregulated after silica treatment, consistent with the trend observed at the lung-tissue level, suggesting that fibroblasts are the most likely target cells for NMN to exert its protective effects. Then, we compared the transcriptomic results for the lung tissue and fibroblasts and found that the metabolism of xenobiotics by cytochrome P450 and the glutathione metabolic pathway changed significantly at the tissue level, and also changed significantly in fibroblasts. Since NMN alleviated oxidative damage by modulating these signaling pathways at the tissue level, we presumed that NMN would have the same regulatory effect on fibroblasts in the lung-tissue microenvironment. However, it was not further verified whether NMN directly mitigates oxidative damage to fibroblasts in vitro. In silica-induced lung injury, fibroblast dysfunction is not directly caused by silica but the microenvironmental changes caused by other cells, such as the excessive release of cytokines or ROS. However, no research has yet fully analyzed the composition of the lung microenvironment in silicosis, or even in other pulmonary inflammatory and fibrosis diseases. Therefore, it is difficult for us to simulate the microenvironment and the oppression of fibroblasts when exposed to silica in vitro, hindering us from using NMN for intervention with fibroblasts alone in order to determine whether NMN can improve lung injury by regulating the expression of *Nfe2l2*, *Gstm1*, *Gstm1* and *Mgst1* in fibroblasts, and thus more research is needed.

## 5. Conclusions

In summary, our study found that silica exposure causes oxidative damage to the lungs. NMN regulates the cytochrome P450-mediated antioxidant signaling pathway and the metabolism of glutathione by promoting the nuclear transfer of Nrf2 and upregulating the expression of *Gstm1*, *Gstm2*, and *Mgst1*, in turn promoting the production of GSH and scavenging ROS, thereby alleviating silica-induced lung injury. Moreover, fibroblasts may be the target cells for NMN to exert antioxidant effects by regulating cytochrome P450 and the glutathione metabolism pathway. Our study reveals that NMN may be a promising candidate for adjuvant silicosis therapy.

## Figures and Tables

**Table 1:** Animal experiment design scheme.

Groups	Intratracheal Instillation (0 d)	i.g. * (Daily)	Number of Animals (7 d)	Number of Animals (28 d)
Control group (Sham)	Normal saline (80 μL)	Normal saline	3	5
Model group (Vehicle)	Silica (50 mg/mL, 80 μL)	Normal saline	5	6
NMN high-dose group (NMN-H)	Silica (50 mg/mL, 80 μL)	NMN (1000 mg/kg)	5	6
NMN low-dose group (NMN-L)	Silica (50 mg/mL, 80 μL)	NMN (500 mg/kg)	5	6
NMN control group (NMN-Con)	Normal saline (80 μL)	NMN (1000 mg/kg)	3	5

* i.g.: Intragastric administration.

**Table 2:** qRT-PCR primers used in this experiment.

Primer Name	Forward Primer	Reverse Primer
Gstm1	CCATTGCCAAACCCTTTGCT	TGACCTTGTCCCCTGCAAAC
Mgst1	GCTCGGATCTACCACACCATTGC	CTCCTTAGCAGCCTGTAAGCCATTG
Gstm2	GAGAGACAGAGGAGGAGAGGATTCG	TCTCAAAGTCAGGGCTGTAGCAAAC
Nfe2l2	AAGCACAGCCAGCACATTCTCC	TGACCAGGACTCACGGGAACTTC
Gsto1	GCCCGAGTGGTTCTTTGAGA	GTGCCTTCTTGTACGGGTCA
Gsta4	TGTATGGGAAGGACCTGAAGGAGAG	ATGGAGCCACGGCAATCATCATC
β-actin	GAGGTATCCTGACCCTGAAGTA	CACACGCAGCTCATTGTAGA

## Data Availability

The data that support the findings of this study are available from the corresponding author upon reasonable request.

## Supplementary Materials

The following supporting information can be downloaded at: https://www.mdpi.com/article/10.3390/nu15010143/s1. File S1: NMN purity assay; Figure S1: Effects of silica exposure and NMN supplementation on *Gsta4* and *Gsto1* expression in lung tissue; Figure S2: The expression of *Gstm1*, *Gstm2*, *Mgst1*, and *Nfe2l2* in lung tissue after silica exposure; Figure S3: The expression of *Gstm1*, *Gstm2*, *Mgst1*, and *Nfe2l2* in fibroblasts after silica exposure; Table S1: The top 10 GO terms of biological process, cellular component, and molecular function; Table S2: KEGG enrichment analysis of 30 differential genes.
