# Supplementary material for: Nicotinamide Mononucleotide Ameliorates Silica-Induced Lung Injury through the Nrf2-Regulated Glutathione Metabolism Pathway in Mice
Source: Nutrients. 2022 Dec 28;15(1):143. doi: 10.3390/nu15010143 (PMC9823503; doi:10.3390/nu15010143)
Supplement: Supplementary file 1 [file nutrients-15-00143-s001.zip › Revised Supplementary Figure-nutrients.pdf]

Supplementary Figures

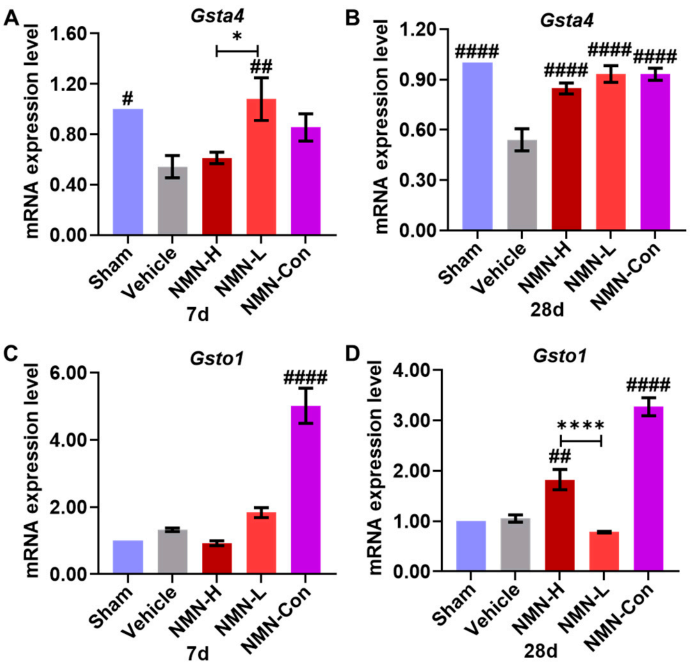

Supplementary Figure S1. Effects of silica exposure and NMN supplementation on *Gsta4* and *Gsto1* expression in lung tissue.

(**A**) The relative expression level of *Gsta4* mRNA in lung tissue at 7 d; (**B**) relative expression level of *Gsta4* mRNA in lung tissue at 28 d; (**C**) relative expression level of *Gsto1* mRNA in lung tissue at 7d; (**D**) relative expression level of *Gsto1* mRNA in lung tissue at 7d. (# means $P < 0.05$,## means $P < 0.01$, #### means $P < 0.0001$ vs Vehicle; * means $P < 0.05$, **** means $P < 0.0001$, NMN-H vs NMN-L). Sham: saline + saline; Vehicle: silica + saline; NMN-H: silica + NMN (1000 mg/kg); NMN-L: silica + NMN (500 mg/kg); NMN-Con: saline + NMN (1000 mg/kg).

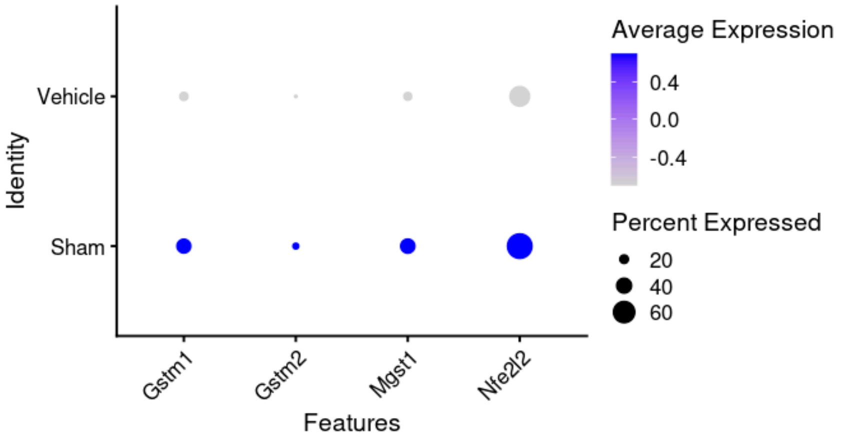

Supplementary Figure S2. The expression of *Gstm1*, *Gstm2*, *Mgst1* and *Nfe2l2* in lung tissue after silica exposure.

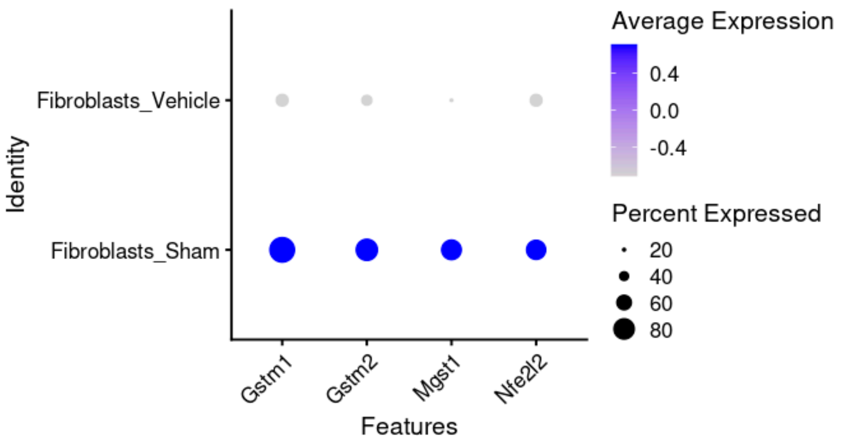

Supplementary Figure S3. The expression of *Gstm1*, *Gstm2*, *Mgst1* and *Nfe2l2* in fibroblasts after silica exposure.
