# Supplementary material for: Nicotinamide Mononucleotide Ameliorates Silica-Induced Lung Injury through the Nrf2-Regulated Glutathione Metabolism Pathway in Mice
Source: Nutrients. 2022 Dec 28;15(1):143. doi: 10.3390/nu15010143 (PMC9823503; doi:10.3390/nu15010143)
Supplement: Supplementary file 1 [file nutrients-15-00143-s001.zip › Revised Supplementary Materials File S1.pdf]

Supplementary Materials File S1: NMN purity assay

NMN detection procedure

Sample solution: 0.5 mg/mL of NMN in Diluent.

Solution A: 6.8 g/L of potassium dihydrogen phosphate in water.

Solution B: Methanol.

Diluent: Water.

Mobile phase: See below.

Time(min)	Solution A (%)	Solution B (%)
0	100	0
10	100	0
20	50	50
21	100	0
29	100	0

Chromatographic system

Mode: LC

Detector: UV 260nm

Column: WelchUltimate AQ-C18 4.6-mm × 25-cm; 5-µm

Column temperature: 30℃

Flow rate: 0.8 mL/min

Injection volume: 10 µL

Chromatograms and results

Injection infromation

Injection name: NMN
Bottles No: B:A5
Injection type: N/A
Correction Level:
Instruments methods: Determination of NMN related substances 1102
Processing method: 3D quantify
Injection date and time: 2022/05/06 14:27

Run time: 40.00
Injection volum: 10.00
Channel: UV_VIS_1
Wavelength: 260.0
Bandwidth: 4
Dilution factor: 1.0000
Sample weight: 1.0000

Chromatograms

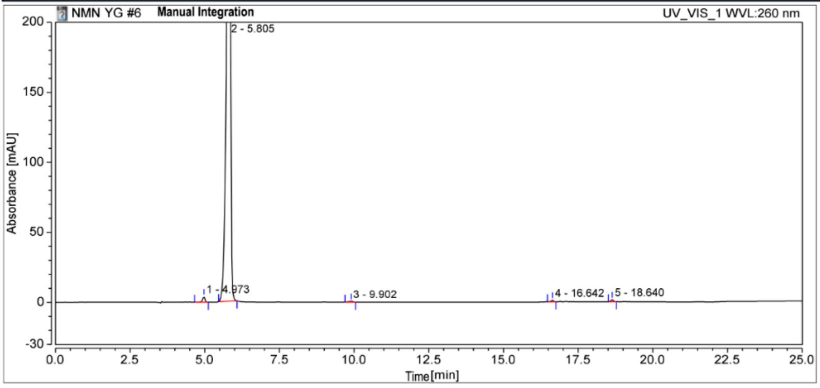

Integral result

Serial number	Peak name	Retention Time	Relative peak area mAU*min	Peak height mAU	Relative peak area %	Relative peak height %	Sample volume
1		4.973	0.356	4.151	0.37	0.45	n.a.
2		5.805	96.341	919.308	99.27	99.17	n.a.
3		9.902	0.096	0.596	0.10	0.06	n.a.
4		16.642	0.116	1.315	0.12	0.14	n.a.
5		18.640	0.136	1.589	0.14	0.17	n.a.
Total			97.045	926.959	100.00	100.00	
